# Effects of red clover isoflavones on tall fescue seed fermentation and microbial populations *in vitro*

**DOI:** 10.1371/journal.pone.0201866

**Published:** 2018-10-18

**Authors:** Emily A. Melchior, Jason K. Smith, Liesel G. Schneider, J. Travis Mulliniks, Gary E. Bates, Zachary D. McFarlane, Michael D. Flythe, James L. Klotz, Jack P. Goodman, Huihua Ji, Phillip R. Myer

**Affiliations:** 1 Department of Animal Sciences, University of Tennessee, Knoxville, TN, United States of America; 2 Department of Plant Sciences, University of Tennessee, Knoxville, TN, United States of America; 3 USDA-ARS, Forage-Animal Production Research Unit, Lexington, KY, United States of America; 4 Department of Plant and Soil Sciences, University of Kentucky, Lexington, KY, United States of America; 5 College of Agriculture, Food and Environment, University of Kentucky, Lexington, KY, United States of America; The University of Sydney, AUSTRALIA

## Abstract

Negative impacts of endophyte-infected *Lolium arundinaceum (Darbyshire)* (tall fescue) are responsible for over $2 billion in losses to livestock producers annually. While the influence of endophyte-infected tall fescue has been studied for decades, mitigation methods have not been clearly elucidated. Isoflavones found in *Trifolium pratense* (red clover) have been the subject of recent research regarding tall fescue toxicosis mitigation. Therefore, the aim of this study was to determine the effect of ergovaline and red clover isoflavones on rumen microbial populations, fiber degradation, and volatile fatty acids (VFA) in an *in vitro* system. Using a dose of 1.10 mg × L^-1^, endophyte-infected or endophyte-free tall fescue seed was added to ANKOM fiber bags with or without 2.19 mg of isoflavones in the form of a control, powder, or pulverized tablet, resulting in a 2 × 3 factorial arrangements of treatments. Measurements of pH, VFA, bacterial taxa, as well as the disappearance of neutral detergent fiber (aNDF), acid detergent fiber (ADF), and crude protein (CP) were taken after 48 h of incubation. aNDF disappearance values were significantly altered by seed type (*P* = 0.003) and isoflavone treatment (*P* **=** 0.005), and ADF disappearance values were significantly different in a seed × isoflavone treatment interaction (*P* ≤ 0.05). A seed × isoflavone treatment interaction was also observed with respect to CP disappearance (*P* ≤ 0.05). Eighteen bacterial taxa were significantly altered by seed × isoflavone treatment interaction groups (*P* ≤ 0.05), eight bacterial taxa were increased by isoflavones (*P* ≤ 0.05), and ten bacterial taxa were altered by seed type (*P* ≤ 0.05). Due to the beneficial effect of isoflavones on tall fescue seed fiber degradation, these compounds may be viable options for mitigating fescue toxicosis. Further research should be conducted to determine physiological implications as well as microbiological changes *in vivo*.

## Introduction

Ergot alkaloids produced by the endophytic fungus *Epichloë coenophiala* found in the endophyte-infected tall fescue plant (*Lolium arundinaceum Schreb*.) contribute to drought, heat, and disease tolerance of the plant, but are implicated in expensive production losses when grazed by livestock [1]. Concerns of the alkaloids manifest with reduced blood flow to the periphery, reduced reproductive efficiency, and reductions in growth performance [2–4]. Of the alkaloids associated with endophyte-infected tall fescue, ergovaline is the most predominant of concern in the plant, as it imposes the greatest detrimental effects on livestock [5, 6]. Reducing the consumption of endophyte-infected tall fescue has proven to be a challenge for livestock producers, as it occupies roughly 15 million hectares in the United States [7], with the majority in the east and southeast regions of the country. Replacing endophyte-infected varieties with novel and endophyte-free varieties of tall fescue can abate toxicosis, but may not be a cost-effective long-term solution [8]. Alternatively, the incorporation of cool-season legumes into endophyte-infected tall fescue pastures has been shown to mitigate a portion of tall fescue toxicosis symptoms [9, 10]. Phytoestrogenic compounds found in red and white clover have been reported to improve overall growth performance of livestock grazing endophyte-infected tall fescue throughout the spring and summer months [11–13]. Various isoflavones have been targeted for use in human medicine due to their beneficial reduction in breast cancer symptoms, improvements in cardiovascular health, as well as reductions in menopausal symptoms [14–16]. As such, determining additional benefits of isoflavones, such as the improvement of fiber utilization and rumen fermentation in livestock, is timely.

Ergot alkaloid pressure, or the physiological burden, is reduced in the presence of ruminal fluid [17, 18], indicating that ruminal microorganisms may be responsible for degradation of ergot alkaloids to less harmful compounds such as lysergic acid. Harlow, Goodman (19) identified several hyper-ammonia producing and tryptophan-utilizing bacteria capable of degrading ergovaline. Tryptophan is essential for ergovaline synthesis and formation of the ergoline ring structure. It has been hypothesized that bacterial species able to degrade tryptophan may also degrade ergovaline in the rumen [19, 20], proving essential for reducing the effects of tall fescue toxicosis. What is not as clear is the effect of ergovaline on the ruminal microbiome. As diet and toxins effect ruminal fermentation and production of glucogenic precursors required for the nutrition of the animal [21, 22], it is critical to determine the extent to which endophyte toxins in fescue may impact microbial numbers, alter microbial profiles, or influence volatile fatty acid (VFA) production and concentrations. While a phylogenetically [23] and functionally diverse rumen microbiome is essential to the health and nutritional status of ruminant animals, feed additives or plant-secondary compounds, such as isoflavones, may further improve rumen functionality in tandem with microbial populations. Isoflavones may enhance rumen function by improving fiber degradation and by reducing several microorganisms responsible for lowered ruminal pH and increased lactate production that can disrupt normal functional microflora [24, 25].

This study examined the effect of endophyte-infected or endophyte-free tall fescue seed with or without an isoflavone source on *in vitro* rumen fermentation, fiber degradation, and rumen bacterial communities. The objectives of this study were to 1) determine if isoflavones improve fiber degradation and fermentation of tall fescue seed *in vitro*, and 2) determine if *in vitro* rumen bacterial populations are affected by seed type or the addition of isoflavones. We hypothesized that the addition of isoflavones to endophyte-infected tall fescue seed would alter *in vitro* fermentation and rumen bacterial populations.

## Materials and methods

### Study design

This study was approved and carried out in accordance with the recommendations of the Institutional Animal Care and Use Committee at the University of Tennessee, Knoxville. Rumen content for the study was acquired from two fistulated Holstein heifers. The cattle are maintained and managed by the standard operating procedures of the Cherokee Farm (Knoxville, TN) as part of the University of Tennessee Institute of Agriculture. Heifers are continually maintained on mixed cool-season grass pastures.

This experiment was conducted as a randomized incomplete block design, and was blocked by each run. Eight separate fermentation runs were performed with the Daisy^II^ incubator (ANKOM Technology Co. Ltd. NY, USA). The first seven runs included four ANKOM fermentation jars (ANKOM Technology Co. Ltd. NY, USA) to which treatments were randomly allocated, and the eighth run consisted of two fermentation jars, for a total of five replications of six treatment combinations. Treatments were arranged as a 3 × 2 factorial design that included a commercial mix of isoflavones: (1) Promensil 80 mg (net) isoflavone tablet, (2) Promensil 80 mg (net) equivalent isoflavone powder, or (3) control, receiving no isoflavones, and two seed types: (1) endophyte-infected tall fescue seed (KY 31) or (2) endophyte-free tall fescue seed (KY 32). All seed was purchased from Turner Seed Inc. (Winchester, KY). Isoflavones in the form of the Promensil product were supplied by PharmaCare Laboratories (Warriewood, NSW 2102, Australia). As the Promensil tablet is processed and bound, the unprocessed powder was also examined.

For the responses of aNDF and ADF disappearance, ten fiber bags per fermentation jar were utilized as the sampling units. This yielded a total number of aNDF and ADF disappearance observations of 300. For the response of CP disappearance, five bags per fermentation jar were composited to a total of two weigh boats per fermentation jar as the sampling unit. This yielded a total number of CP disappearance observations of 60. Similarly, for the responses of ruminal pH and volatile fatty acids (VFA), two samples per fermentation jar were obtained using a 15-mL conical tube, yielding 60 ruminal pH and VFA observations.

### Quantification of ergot alkaloids

Fescue seeds have been utilized in this study to eliminate any issues pertaining to volume and fiber that may impede or confound the interpretation of isoflavone effects on endophyte-infected tall fescue seed fermentation. This method has been utilized by numerous studies under this rationale [26, 27]. Prior to the study, concentrations of ergovaline and its epimer ergovalinine in fescue seed were determined using HPLC with fluorescence detection as described in Aiken et al. [27] with modifications described by Koontz et al. [28]. The endophyte-infected fescue seed contained 2.94 ppm of ergovaline and ergovalinine (1.85 and 1.09 ppm, respectively), and the endophyte-free tall fescue seed contained a total of 0 ppm of ergot alkaloids. Additionally, both seed varieties tested negative for the presence of the alkaloid ergotamine and its epimer ergotaminine. Seed samples were ground through a 5-mm screen using a Wiley Mill before 0.4425g of seed (1.3865 × 10^−6^ g ergot alkaloids) was added to each ANKOM fiber bag.

### Quantification of isoflavones

Isoflavones were procured by finely grinding the product Promensil (PharmaCare Laboratories, Warriewood, NSW 2102, Australia), an over-the-counter isoflavone supplement isolated from red clover, with mortar and pestle to pass through a 1 mm screen. Quantification of isoflavones in Promensil, including biochanin A, formononetin, genistein and daidzein, was performed using methods similar to those previously described by Aiken et al. (2016), using LC-MS rather than UV for detection. Briefly, isoflavone extracts were prepared by adding 7 mL of 85% methanol in 0.5% acetic acid to ground samples in 50-mL conical polypropylene tubes. Samples were briefly vortexed and sonicated for 30 min at ambient temperature. Three mL of deionized water was added to each sample prior to being vortexed and centrifuged for 8 min at 2200 × g. The resulting supernatant was filtered through a 0.45 μm GHP membrane syringe filter. Extracts were then diluted, and flavone added as internal standard. One portion of each sample was analyzed as-extracted and a second portion was heated at 85°C for 5 h to hydrolyze isoflavone malonyl-glucosides to their corresponding isoflavone glucosides. Concentrations of biochanin a-malonyl-glucoside and formononetin-malonyl-glucoside were determined by difference between hydrolyzed and un-hydrolyzed portions. Isoflavone extracts were analyzed by LC-MS on a Waters Acquity UPLC coupled to a Waters Synapt G2 (q-ToF) high resolution mass spectrometer. Chromatographic separation was obtained using a Waters BEH C18 UPLC column (1.7 μm, 2.1 mm x 150 mm). The mobile phase employed a mixture of water containing 0.1% formic acid (solvent A) and acetonitrile containing 0.1% formic acid (solvent B) in a linear gradient from 20% B to 80% B at a flow rate of 0.35 mL x min^-1^. The high resolution mass spectrometer was operated in positive ion electrospray mode with a resolving power of ~14,000 and scanned from 100 to 1000 Da in 0.3 s. Leucine enkephalin was used to provide a lock mass (m/z 554.2615). Quantification of isoflavones was performed using QuanLynx software with a linear calibration curve and internal standard method. Extracted ion chromatograms with a mass window of 0.02 Da around the accurate mass of each analyte were used to calculate peak areas. A total of 2.19 mg of total isoflavones were provided per bag (0.92 mg biochanin A, 0.89 mg formononetin, 0.0038 mg genistein and 0.0016 mg daidzein), for a concentration of 1.10 mg × L^-1^ of rumen fluid and buffer.

### *In vitro* fiber and crude protein disappearance

The Daisy^II^ *in vitro* incubation system (ANKOM Corp., Fairport, NY) was utilized to determine the rate and extent of fiber disappearance of the endophyte-infected or endophyte-free tall fescue seed with or without the addition of isoflavones from Promensil. Each substrate (500 ± 40 mg) was weighed into artificial fiber bags (F57 fiber bags, ANKOM Corp.), which were then heat sealed. Content included 442.5 mg of either endophyte-infected (KY 31) or endophyte-free tall fescue (KY 32) seed (dry matter basis), and 57.5 mg of Promensil (tablet or powder, dry matter basis). The fiber bags were separated into four groups of 12 bags within a seed type x isoflavone treatment combination, including two empty bags for correction, and placed into separate upright plastic containers. A total of 1600 mL of rumen fluid was procured via aspiration from two fistulated Holstein heifers (Cherokee Farm, Knoxville, TN), and buffered using a 1:4 dilution of rumen fluid to McDougall’s artificial saliva buffer [29]. A total of 400 mL were added to each ANKOM fermentation jar with 1600 mL of buffer, and adjusted to a pH of 6.8 with CO_2_. The fiber bags and diluted/buffered rumen fluid were added to the incubation containers. Incubation then proceeded for 48 h at 39 ± 0.5°C.

Upon completion of the 48-h incubation, fermented rumen fluid pH was measured using a pH meter (Denver Instruments, Bohemia, NY) and 15 mL of fermented rumen content was sampled and stored at -80°C until further processing for bacterial DNA [30, 31]. All fiber bags were removed from fermentation jars, and washed under tap water before further analyses. Analysis of aNDF was obtained using α-amylase and sodium sulfite [32], and ADF was determined using sulfuric acid-based detergent [33]. Both procedures were conducted using the ANKOM200 fiber analysis system (ANKOM Corp., Fairport, NY). Crude protein was determined by total nitrogen combustion analysis (LECO Instruments, Inc., St. Joseph, MI). Difference between pre- and post-fermentation aNDF, ADF, and CP concentrations were used to calculate disappearance.

### VFA analysis

A subsample of rumen fermentation fluid was aliquoted from each fermentation jar for VFA analysis using HPLC, similar to the procedures previously described by Harlow et al. (2017). Briefly, samples were analyzed for concentrations of acetate, propionate, butyrate, valerate, and isovalerate/methylbutyrate (IVMB) using a Summit HPLC (Dionex; Sunnyvale, CA, USA) equipped with an anion exchange column (Aminex HP-87H; Bio-Rad, Hercules, CA, USA) and UV detector. Column parameters include a 300 x 7.8 mm, prepacked HPLC carbohydrate analysis column, hydrogen form, 9 μm particle size, 8% cross linkage, with a flow rate of 0.4 mL/min and an injection volume of 100 μL. The eluting compounds were separated isocratically with an aqueous sulfuric acid solution (5 mM). The parameters included an injection volume of 0.1 mL, flow rate of 0.4 mL × min^-1^, and column temperature of 50°C. Isovalerate and methyl butyrate (IVMB) resolve together in a single peak, and consequently, are reported together. A total VFA concentration (mMol) was calculated for each sample by summing the concentrations of all VFAs. A proportion of each specific VFA was calculated from the total by dividing the specific VFA by the total VFA and multiplying by 100 (units = mmol/100mmol total).

### DNA extraction, PCR, and sequencing

DNA was extracted from ruminal fluid post-fermentation. The procedure of the DNA extraction method was similar to that previously described by Yu and Morrison [34]. After the chemical/mechanical cell lysis and isopropanol precipitation of nucleic acids, metagenomic DNA was purified with Rnase and proteinase K treatment, followed by the use of QIAamp columns from the Qiagen DNA Stool Mini Kit (Qiagen, Hilden, Germany). Genomic DNA concentration was determined using a Nanodrop 1000 spectrophotometer (Thermo Scientific, Wilmington, DE), and verified using Invitrogen Qubit fluorometer with PicoGreen (ThermoFisher Scientific, Wilmington, DE). Extractions were stored at -20˚C until sequencing library preparation. Bacterial 16S rRNA genes were PCR-amplified with dual-barcoded primers targeting the V4 region, as per the protocol of Kozich [35]. Amplicons were sequenced with an Illumina MiSeq using the 250-bp paired-end kit (v.2). Sequences were denoised, taxonomically classified using Greengenes (v. 13_8) as the reference database, and clustered into 97% similarity operational taxonomic units (OTUs) with the mothur software package (v. 1.39.5), as previously described by Schloss, Westcott [36], and following the manufacturer-recommended procedure (https://www.mothur.org/wiki/MiSeq_SOP; accessed November 2017). Sequence files are available from the NCBI Sequence Read Archive (SRA Accession SRP150716). Additional descriptive information is associated with NCBI BioProject PRJNA476124.

### Statistical analyses

The efficiency of the incomplete block design [37] was determined using PROC OPTEX in SAS 9.4 (SAS Inst. Inc., Cary, NC). Study analyses included responses of aNDF, ADF, CP disappearance, rumen pH, total VFA concentrations, specific VFA concentration in relation to total, and ruminal bacterial communities. Separate mixed model analyses of variance (ANOVA) were performed using the MIXED procedure of SAS to determine the fixed effects of isoflavone treatment, seed type, and seed type × isoflavone treatment interaction. The ANOVA models also included random effects of run and run × seed type × isoflavone treatment. Differences in least square means were separated using Fisher’s least significant differences. When an interaction between main effects was detected, multiple comparisons between all treatment combinations were adjusted using Tukey’s adjustments. Effects were considered significant at *P* ≤ 0.05, and tendencies declared at *P* > 0.05 and ≤ 0.10.

For rumen bacterial communities, analysis was conducted in the R environment. Alpha diversity was estimated with the Shannon index on raw OTU abundance tables. The significance of diversity differences was tested with an ANOVA. To estimate beta diversity across samples, OTUs were excluded if occurring in fewer than 10% of the samples with a count of less than three and computed Bray-Curtis indices. Beta diversity, emphasizing differences across samples, was visualized using non-metric multidimensional (NMDS) ordination. Variation in community structure was assessed with permutational multivariate analyses of variance (PERMANOVA) with treatment group as the main fixed factor and using 4,999 permutations for significance testing.

## Results

### Efficiency of Incomplete block design

Efficiency is a measurement of goodness of an experimental design [32]. D-efficiency is calculated based on the geometric mean of eigenvalues of the variance matrix [32]. In a perfectly balanced design, D-efficiency will equal 100%. In our study, the treatment D-efficiency was 87.6% for our allocation of treatments to runs.

### aNDF, ADF, and CP disappearance

There was a main effect of seed type on aNDF disappearance (*P =* 0.003, Table 1). On average, there was reduced aNDF disappearance in E+ seed compared to that of E- seed (11.53 ± 0.97% vs. 14.02 ± 0.96%, respectively). Additionally, there was an effect of isoflavone treatment on aNDF disappearance (*P* = 0.0053). Isoflavone powder treatment, isoflavone tablet treatment, and control treatment resulted in 14.28 ± 0.93%, 13.21 ± 0.93%, and 10.82 ± 0.93% disappearance, respectively. There was no difference (*P* = 0.49) in aNDF disappearance between powder or tablet forms of isoflavone, and both forms of isoflavone resulted in significantly increased aNDF disappearance compared to the control.

**Table 1:** Effects of isoflavones and fescue seed type on neutral detergent fiber (aNDFD), acid detergent fiber (ADF) and crude protein (CP) disappearance after rumen fluid incubation (48 h).

Measurement (%)	Isoflavone Treatment^1^						SEM	*P*-value		
	Control		Powder		Tablet					
	Fescue Seed^2^									
	E+	E-	E+	E-	E+	E-		Seed	Isoflavone Treatment	Seed × Isoflavone Treatment
**aNDF**	9.11	12.52	12.72	15.84	12.74	13.68	1.24	0.003	0.005	0.432	
**ADF**	1.72^c^	3.21^bc^	7.01^a^	2.19^c^	1.27^c^	5.91^b^	1.03	0.36	0.009	< 0.001
**CP**	2.44^d^	12.23^a^	6.98^bc^	7.33^bc^	4.01^cd^	9.08^ab^	0.93	< 0.001	0.689	0.0002

^1^Treatment: control, isoflavone powder, or isoflavone pulverized tablet.

^2^Seed type: KY 31 endophyte-infected (E+) tall fescue seed, KY 32 endophyte-free (E-) tall fescue seed.

^abcd^ Within an isoflavone treatment, seed types with like superscripts are not different at *P* = 0.05

ADF disappearance was associated with seed × treatment type interaction (*P* ≤ 0.05, Table 1). Effects of seed type on ADF disappearance was dependent upon isoflavone treatment. Samples that received the powdered isoflavone had greater (*P* = 0.0007) ADF disappearance from endophyte-infected tall fescue seed (7.01 ± 1.04%) when compared to endophyte-free tall fescue seed (2.19 ± 1.04%). However, samples that received the tablet isoflavones had lesser (*P* = 0.001) ADF disappearance from endophyte-infected tall fescue seed (1.27 ± 1.04%) when compared to endophyte-free tall fescue seed (5.91 ± 1.04%). Within the control isoflavone treatment, there was no effect of seed type (*P* = 0.52).

There was a seed × treatment interaction on overall CP disappearance (*P* = 0.0002, Table 1). In the control isoflavone group, reduced CP disappearance was observed with endophyte-infected tall fescue seed type (2.44 ± 0.93%) when compared to endophyte-free fescue seed (12.23 ± 0.93%, *P* < 0.0001). There was no CP disappearance difference between endophyte-infected and endophyte-free seed when the powder isoflavone was used (*P* = 0.99); however, when the tablet isoflavone form was supplemented, endophyte-infected tall fescue seed yielded decreased CP disappearance (4.01 ± 0.99%) compared to endophyte free tall fescue seed (9.08 ± 0.93%, *P* = 0.01, Table 1).

### Rumen fermentation fluid VFA concentrations, bacterial populations, and pH

There was a tendency for an effect of seed type on total VFA concentrations. Total VFA was slightly increased in endophyte infected seed (21.68 ± 2.18) compared to endophyte-free seed (18.63 ± 2.1, *P* = 0.06). Propionate concentrations tended to be reduced for endophyte-free tall fescue seed (31.11 ± 1.96) when compared to endophyte-infected seed (33.54 ± 1.96, *P* = 0.075; Table 2), but no effect of treatment or treatment × seed interaction was observed (*P* > 0.1). The acetate: propionate ratio tended to be reduced (*P* = 0.09; Table 2) with endophyte-infected tall fescue seed (1.74 ± 0.15) when compared to endophyte-free tall fescue seed (1.98 ± 0.15). No effects of seed type or isoflavone treatment were observed among acetate, butyrate, valerate or IVMB concentrations (*P* > 0.1).

**Table 2:** Effects of isoflavones and fescue seed type on volatile fatty acid concentrations in the fluid phase of an *in vitro* rumen fluid digestion (48 h).

VFA		Isoflavone Treatment^1^						SEM	*P*-value		
		Control		Powder		Tablet					
		Fescue Seed^2^									
		E+	E-	E+	E-	E+	E-		Seed	Isoflavone Treatment	Seed × Isoflavone Treatment
**Total VFA**^**3**^ **VFA**^**4**^		23.55	20.00	19.66	17.98	21.84	17.90	2.73	0.06	0.33	0.86
			**Acetate**	55.22	59.29	57.85	58.85	56.38	58.39	2.08	0.14	0.82	0.73
	**Propionate**	35.24	31.23	31.15	30.78	34.23	31.30	2.38	0.07	0.34	0.56
	**Butyrate**	7.94	7.88	9.58	8.06	7.07	8.81	1.12	0.95	0.52	0.28
	**Valerate**	1.48	0.52	0.71	1.16	0.91	1.30	0.50	0.91	0.92	0.24
	**IVMB**^5^	0.81	1.05	0.76	0.81	0.75	0.84	0.24	0.15	0.35	0.68
	**Acetate:Propionate**	1.60	1.97	1.90	1.99	1.73	1.98	0.20	0.09	0.62	0.73

^1^Treatment: control, isoflavone powder, or isoflavone pulverized tablet.

^2^Seed type: KY 31 endophyte-infected (E+) tall fescue seed, KY 32 endophyte-free (E-) tall fescue seed.

^3^ Concentration in mMol

^4^ Concentration in mMol×100mMol^-1^.

^5^IVMB: Isovalerate/methylbutyrate

**Fig 1:**
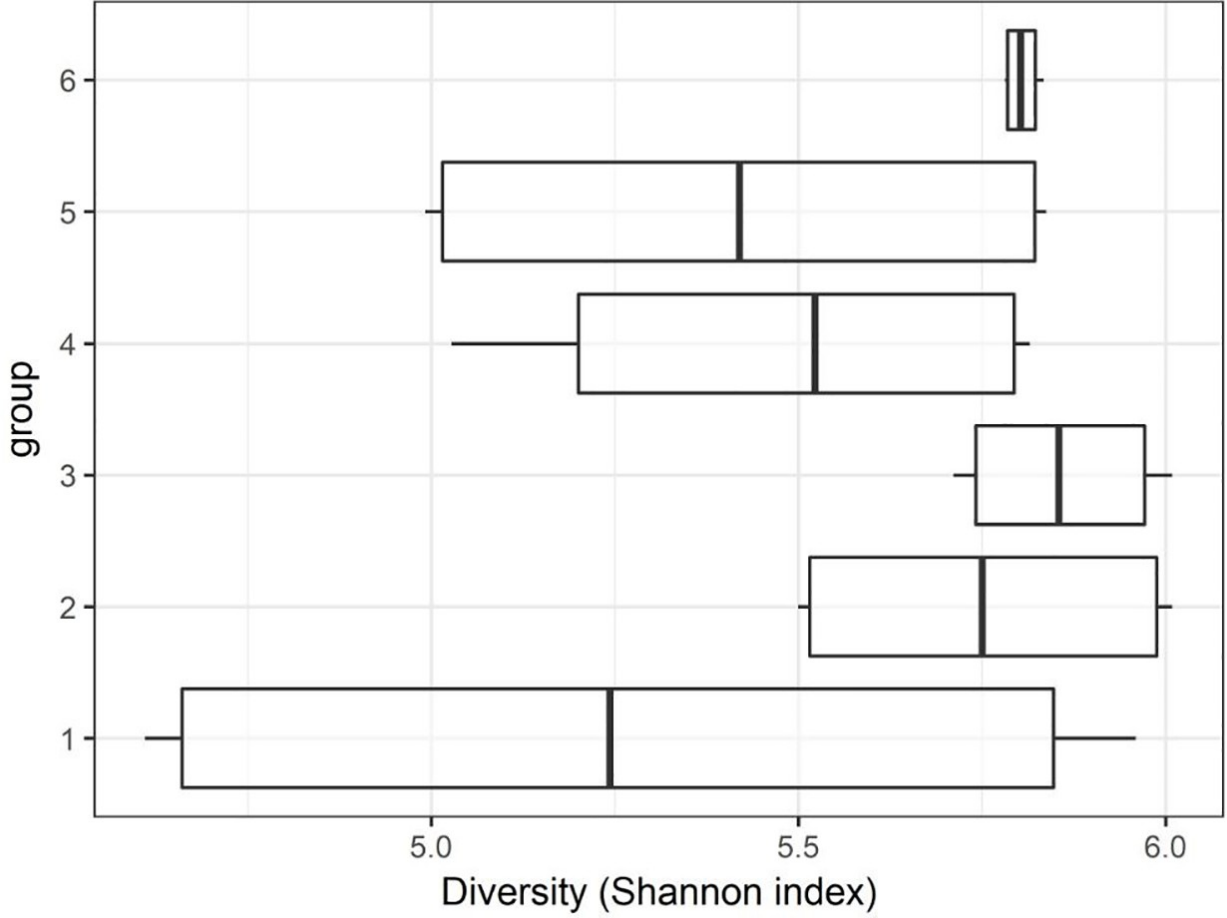
Shannon’s Diversity Index box plot of bacterial species diversity across treatment groups. Group 1: endophyte-infected tall fescue seed. Group 2: endophyte-free tall fescue seed. Group 3: endophyte-infected tall fescue seed with isoflavone powder. Group 4: endophyte-infected tall fescue seed with isoflavone tablet. Group 5: endophyte-free tall fescue seed with isoflavone powder. Group 6: endophyte-free tall fescue seed with isoflavone tablet.

**Fig 2:**
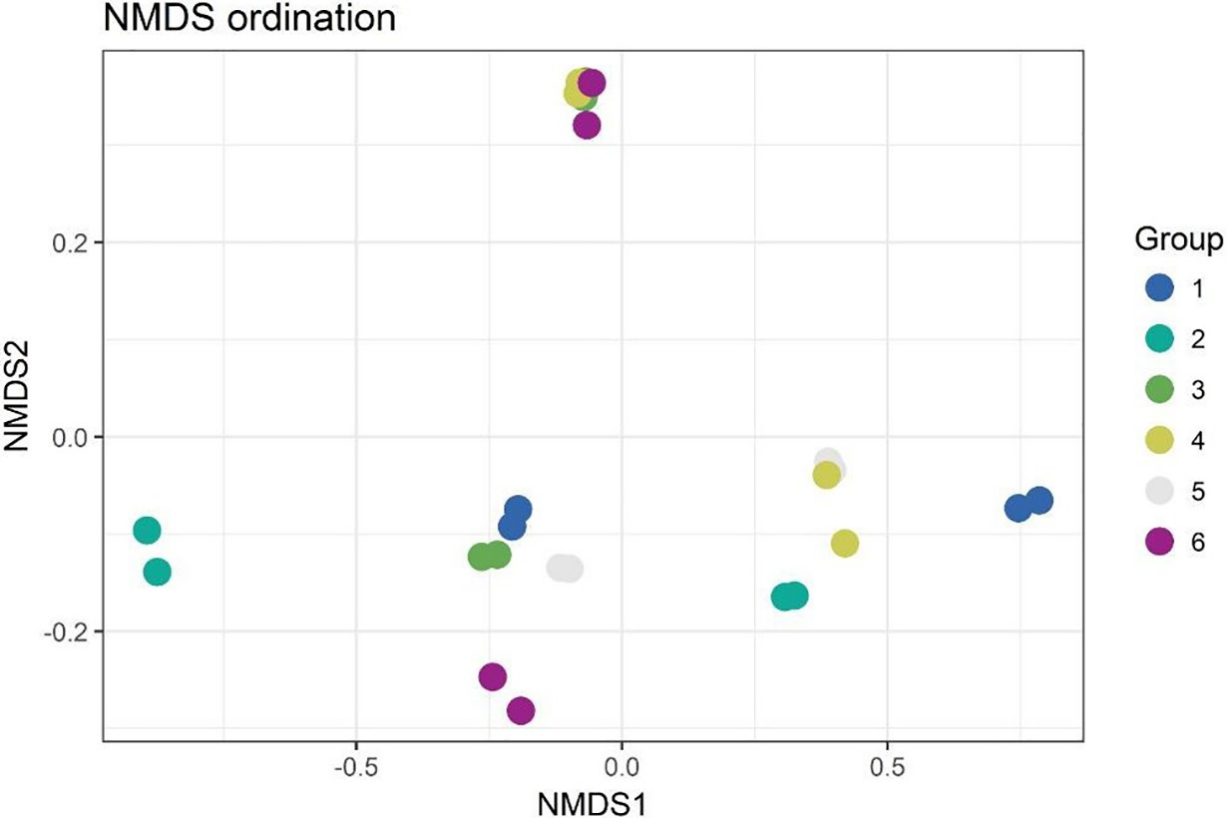
NMDS ordination plot grouped by treatment. Points represent taxa within treatment groups. Similar clustering indicates similar taxa within treatment groups. Group 1: endophyte-infected tall fescue seed. Group 2: endophyte-free tall fescue seed. Group 3: endophyte-infected tall fescue seed with isoflavone powder. Group 4: endophyte-infected tall fescue seed with isoflavone tablet. Group 5: endophyte-free tall fescue seed with isoflavone powder. Group 6: endophyte-free tall fescue seed with isoflavone tablet.

**Fig 3:**
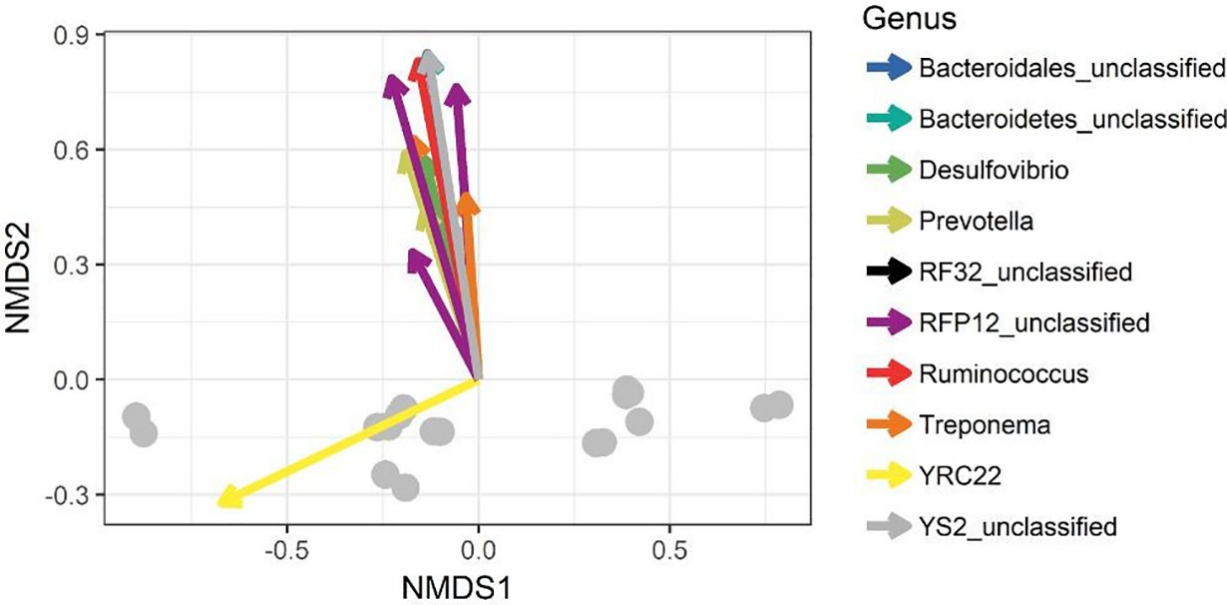
NMDS ordination plot indicating significant taxa at the genus level that influenced major bacterial shifts among treatments.

**Fig 4:**
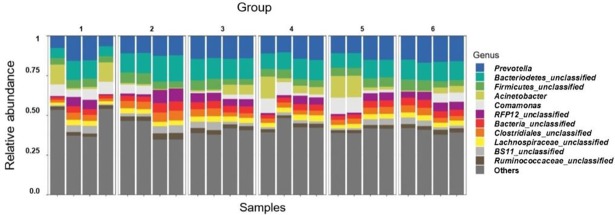
Taxonomic profiles of the relative proportions of bacterial communities by genus, grouped by treatment. Group 1: endophyte-infected tall fescue seed. Group 2: endophyte-free tall fescue seed. Group 3: endophyte-infected tall fescue seed with isoflavone powder. Group 4: endophyte-infected tall fescue seed with isoflavone tablet. Group 5: endophyte-free tall fescue seed with isoflavone powder. Group 6: endophyte-free tall fescue seed with isoflavone tablet. No significant differences among groups (*P* > 0.1).

After stringent sequence processing, a total of 408,813 high quality reads were obtained and averaged 17,034 ± 3,180 reads per sample, which is a consistent depth for sequencing analyses from ruminal samples [38]. Number of observed OTUs totaled 48,867 and averaged 2,037 ± 169 per sample. PERMANOVA global pairwise comparisons indicated significant differences among treatment groups (*P* = 0.03, *R*^*2*^ = 0.32), however Shannon’s Diversity Index was not affected by treatment group (*P* > 0.1, Fig 1). Non-metric multidimensional scaling (NMDS) was utilized to analyze beta diversity (Figs 2 and 3), where clusters of samples represent similarity of 16S rRNA bacterial genera by group based on rank. While treatment groups may have appeared to cluster similarly, there was no observable pattern between groups. Relative proportions of the genus level diversity between groups are represented in Fig 4. No differences in taxonomic profile were observed among groups (*P* > 0.1). However, finer changes in OTU abundances were identified. Numerous taxa were significantly different by seed, treatment, or seed × treatment interaction (*P* ≤ 0.05; Table 3). Fermented rumen fluid pH was not significantly affected by seed type, treatment or seed × treatment interaction (*P* > 0.1), and pH ranged from 6.97 to 7.57.

**Table 3:** Effects of isoflavones and fescue seed type on relative abundance of significant *in vitro* rumen bacterial taxa.

Classification	Percentage of total sequences^1^						Effect	SEM	*P*-value^4^
	Treatment^2^								
	Control		Powder		Tablet				
	Fescue Seed^3^								
	E+	E-	E+	E-	E+	E-			
**Actinomycetales_unclassified**	0.0011^b^	ND^b^	0.013^ab^	0.0027^b^	0.012^ab^	0.019^a^	Treatment	0.0047	0.018
**Alphaproteobacteria_unclassified**	0.012^bc^	0.017^abc^	0.0255^ab^	ND^c^	0.0203^abc^	0.037^a^	Seed × Treatment	0.0071	0.020
***Anaeroplasma***	0.054^ab^	0.067^ab^	0.0233^b^	0.075^ab^	0.044^b^	0.108^a^	Seed × Treatment	0.019	0.037
***Anaerovibrio***	0.074^bc^	0.014^c^	0.244^a^	0.104^bc^	0.158^ab^	0.096^bc^	Seed	0.0447	0.027
***Arcobacter***	0.928^bc^	0.277^c^	1.44^b^	2.34^a^	1.39^b^	0.574^bc^	Seed × Treatment	0.2937	0.015
**Bacteria_unclassified**	4.06^b^	5.56^a^	5.54^a^	4.16^ab^	4.23^ab^	5.49^ab^	Seed × Treatment	0.4920	0.015
**Bacteroidetes_unclassified**	8.77^d^	13.96^a^	12.62^ab^	10.11^cd^	11.26^bc^	11.58^abc^	Seed × Treatment	0.8247	0.001
***Bulleidia***	0.079^b^	0.21^a^	0.11^b^	0.12^b^	0.086^b^	0.096^b^	Seed × Treatment	0.0219	0.019
**Christensenellaceae_unclassified**	0.81^b^	1.61^a^	0.742^b^	0.801^b^	0.721^b^	0.941^b^	Seed	0.1092	0.031
**Clostridiales_unclassified**	3.33^b^	5.60^a^	4.18^ab^	3.92^b^	3.22^b^	3.98^b^	Seed × Treatment	0.2961	0.039
***Corynebacterium***	0.041^c^	0.065^bc^	0.131^a^	0.109^ab^	0.066^bc^	0.076^bc^	Seed	0.0014	0.009
**Endomicrobia_unclassified**	ND^b^	0.0115^a^	ND^b^	0.0015^b^	0.0015^b^	0.0056^ab^	Seed × Treatment	0.0024	0.007
**Gammaproteobacteria_unclassified**	0.018^cd^	0.06^ab^	0.056^abc^	0.013^d^	0.043^bcd^	0.083^a^	Seed × Treatment	0.0152	0.006
***Megasphaera***	0.117^a^	0.018^b^	0.0411^b^	0.0431^b^	0.0344^b^	0.0286^b^	Seed × Treatment	0.0502	0.008
***Methylobacillus***	ND	0.0014	0.0043	0.0042	ND	ND	Treatment	0.0051	0.020
**ML615J.28_unclassified**	0.027^b^	0.065^b^	0.109^b^	0.123^b^	0.123^b^	0.456^a^	Treatment	0.3030	0.009
***Moraxella***	0.0092^ab^	0.0012^b^	0.0142^ab^	0.0029^b^	0.027^a^	0.0088^ab^	Seed	0.0038	0.030
***Olsenella***	0.0292^b^	0.148^a^	0.053^b^	0.038^b^	0.034^b^	0.039^b^	Seed × Treatment	0.0233	0.021
***p*.*75*.*a5***	0.028^c^	0.103^a^	0.047^bc^	0.071^ab^	0.063^abc^	0.071^ab^	Seed	0.0134	0.004
***Peptoniphilus***	0.0069^ab^	ND^b^	ND^b^	0.0146^a^	0.0063^ab^	0.0044^b^	Seed × Treatment	0.0033	0.011
**Peptostreptococcaceae_unclassified**	0.0578^a^	0.0088^c^	0.0024^c^	0.0278^bc^	0.0373^ab^	0.0162^bc^	Seed × Treatment	0.0094	0.003
**Pirellulaceae_unclassified**	0.066^b^	0.196^a^	0.0811^b^	0.125^ab^	0.0677^b^	0.103^ab^	Seed	0.0192	0.019
**Planococcaceae_unclassified**	0.095^a^	0.0014^b^	0.0044^b^	0.0118^b^	0.007^b^	0.0136^b^	Seed × Treatment	0.0168	0.010
***Proteiniclasticum***	1.45^ab^	0.223^c^	2.25^a^	1.29^b^	1.27^b^	1.35^ab^	Seed	0.1770	0.011
***Proteiniphilum***	0.097^ab^	0.027^ab^	0.0203^ab^	0.0069^b^	0.124^a^	0.0148^b^	Seed	0.0203	0.037
**Proteobacteria_unclassified**	0.157^b^	0.196^b^	0.272^ab^	0.192^b^	0.263^ab^	0.378^a^	Treatment	0.0332	0.021
***Pseudobutyrivibrio***	0.496^bc^	0.237^c^	1.11^ab^	0.831^abc^	1.24^a^	0.681^abc^	Treatment	0.1568	0.020
**Ruminococcaceae_unclassified**	2.18^cd^	3.45^a^	2.75^b^	2.04^d^	2.20^cd^	2.66^bc^	Seed × Treatment	0.1852	0.002
***Schwartzia***	0.0334^bc^	0.0087^c^	0.0766^ab^	0.079^a^	0.065^ab^	0.060^ab^	Treatment	0.0108	0.004
***Selenomonas***	0.219^bc^	0.159^c^	0.459^abc^	0.629^a^	0.543^ab^	0.514^ab^	Treatment	0.0842	0.012
**SR1_unclassified**	0.230^bc^	0.0901^c^	0.903^ab^	0.950^a^	0.954^a^	0.607^abc^	Treatment	0.236	0.0104
***Succiniclasticum***	0.672^b^	1.67^a^	0.662^b^	0.774^b^	0.627^b^	0.632^b^	Seed × Treatment	0.191	0.0349
***Succinivibrio***	0.002^b^	0.014^a^	ND^b^	0.004^ab^	0.0015^b^	0.0088^ab^	Seed	0.1670	0.027
**Tenericutes_unclassified**	1.21^cd^	1.01^d^	2.43^a^	1.32^bcd^	2.14^ab^	2.07^abc^	Seed	0.2967	0.008
***Treponema***	0.0078^a^	ND^b^	ND^b^	0.0028^b^	ND^b^	0.0031^b^	Seed × Treatment	0.0011	< 0.001
**Wautersiella**	0.445^b^	0.797^b^	0.865^ab^	0.709^b^	0.722^b^	1.31^a^	Seed × Treatment	0.153	< 0.001

^1^ Data reported as LSMeans

^2^ Treatment: control, isoflavone powder, or isoflavone pulverized tablet

^3^ Seed type: KY 31 endophyte infected (E+) tall fescue seed, KY 32 endophyte-free (E-) tall fescue seed

^4^Within a row, differences between treatment groups are considered significant at *P* ≤ 0.05, and trending toward significance at *P* ≤ 0.10

^abcd^ Different means separation letters within a row indicate significant differences among LSMeans

## Discussion

Previous research conducted by Flythe and Kagan [39] and Harlow et al. [25] that noted improved fiber degradation and ammonia reduction employed biochanin A as the primary isoflavone (30 mg × L^-1^). The present study observed similar improvements in fiber degradation, including only 920 μg biochanin A per fiber bag (0.46 mg × L^-1^) and a total isoflavone concentration of 1.10 mg × L^-1^. The results from this experiment were consistent with those achieved by Harlow et al [25], however the amount of total isoflavones (1.10 mg × L^-1^) in this experiment may have been insufficient to elucidate all of the beneficial effects on fiber degradation and ammonia reduction that were previously reported.

The increase in ADF disappearance observed in the present study is consistent with the observations of Harlow [25], when the inclusion of the isoflavone biochanin A reduced overall ADF values post fermentation compared to the controls. As acid detergent fiber measures the amount of cellulose, which is a relatively indigestible fiber component, the inclusion of isoflavones with tall fescue seed in the present study increased indigestible fiber disappearance. Acid detergent fiber has an inverse relationship with digestible energy, and thus a high ADF component indicates less digestible energy available to the animal. In the present study, isoflavones resulted in a higher ADF disappearance, indicating possible improvement in available energy from tall fescue seed.

The results of CP disappearance in the present study are inconsistent with most tall fescue and CP literature, where proteolytic bacteria, including several species of hyper-ammonia producing bacteria (HAB), are more readily able to degrade ergot alkaloid compounds [19] such as those found in endophyte-infected tall fescue. While these hyper-ammonia bacteria are able to degrade ergovaline, they are a source of inefficiency in the animal when dietary nitrogen is lost as ammonia. Antimicrobials, including ionophores and similar compounds, may be employed in the diet to combat this loss of nitrogen, selectively inhibiting some of the microbiota and reducing wasteful byproducts of digestion [40–42]. Flythe et al. [39, 43] examined red clover isoflavones as antimicrobials due to their resistance to amino-acid degradation and action on HAB. They determined that biochanin A inhibited growth of *Clostridium stricklandii*, a member of HAB, and may prevent amino acid fermentation. A seed × treatment interaction affected CP disappearance, and in general, increased disappearance was observed when endophyte-free tall fescue seed was fed while either no isoflavones were supplemented or when isoflavone was given in tablet form. However, the isoflavone concentration used in the present study may not have provided a sufficient amount of isoflavones to elicit a sizable reduction in ruminal amino acid degradation.

The donor animals (n = 2) used for rumen fluid procurement were not tall fescue-naïve animals, and consumed a forage-based diet. Thus, rumen fluid obtained for this experiment may have been previously exposed to ergot alkaloids, which may have affected the results of ruminal VFA concentrations. Additionally, ruminal microbial populations may have adapted or shifted from previous exposure to ergot alkaloids. The lack of significant differences in overall VFA concentrations (Table 2) provides support that the treatments did not inhibit or improve *in vitro* rumen fermentation. Richards and colleagues [44] similarly described no differences in rumen pH or VFA concentrations when steers consumed endophyte-infected tall fescue with or without soybean hulls. Rumen fermentation fluid pH samples were not below 6.0 across replications of the experiment indicating fiber digestion and cellulolytic bacteria function [45] was not reduced, as expected in the buffered system.

Overall, fiber degradability and VFA concentrations were notably reduced in the study in contrast to forage fermentation studies [46]. This was due to the fermentable substrate, as this study aimed to interrogate the effects of isoflavone supplementation on endophyte-infected tall fescue seed fermentation. Other studies have noted the benefits of utilizing ground tall fescue seed as a means of specifically examining the effects of ergot alkaloid exposure [26, 47–49]. Consistent with the previous studies, the current experiment reflects similar biologically significant results, including those regarding fiber degradation [25, 39], which support the conclusion that isoflavones may be effective at improving fiber utilization when supplemented. Although VFA concentrations were unexpectedly lower contrasted to *in vivo* studies [50, 51], several studies using *in vitro* fermentation have noted reduced concentrations using similar methodologies [24, 52].

Although diet imposes great variability on the bacterial community in the rumen [53], no large changes in bacterial community composition or phylogenetic diversity were identified among treatments. Diet similarity may contribute to these observations, as tall fescue seed-types were not drastically different among treatments and endophyte inclusion may induce smaller population changes. Indeed, rather than large changes in community composition, changes in specific taxa occurred, which may impact ruminal function and/or be impacted by supplementation of isoflavones. Overall, 36 taxa were significantly different by seed, treatment, or seed × treatment interaction (Table 3). Studies by Harlow and colleagues [24] observed several species of amylolytic microbes that may be inhibited by biochanin A: *Streptococcus bovis JB1*, *Streptococcus bovis HC5*, *Lactobacillus reuteri*, and *Selenemonas ruminatium*. Additionally Harlow et al. [25] indicated several species of cellulolytic bacteria that are sensitive to biochanin A: *Fibrobacter succinogenes S85*, *Ruminococcus flavefaciens 8*, and *Ruminococcus albus 8*. In the current study, isoflavone supplementation was demonstrated to reduce Clostridiales with the E- seed, supporting results by Flythe et al. [39] where biochanin A inhibited growth of specific HAB Clostridium species, potentially preventing amino acid fermentation. Other cellulolytic and proteolytic bacteria also significantly varied among the treatments, including the genus *Megasphaera*, which was reduced with isoflavone supplementation. As a major lactic acid utilizer in the rumen, *Megasphaera elsdenii* has positive relationship with *S*. *bovis*, due to lactate production from *S*. *bovis* [54, 55]. Although no *S*. *bovis* variations were identified in the current study, the reduction in *M*. *elsdenii* hints at similar inhibitions noted by other researchers [24]. In contrast, the same species shifts that occurred in studies conducted by Harlow et al. [24] were not observed in the present study. Rather than being inhibited, *Selenemonas* proportions were increased when supplemented with isoflavones. However, in both studies conducted by Harlow et al. [24, 25], biochanin A was the only isoflavone supplemented and thus subsequent studies should be completed to verify population shifts with other isoflavones.

The combined results of this study indicated fermentation modifications which occurred due to treatments, specifically in aNDF, ADF, and CP disappearance, but these results may have been affected by several factors. Where both fiber and crude protein disappearance results were not consistent with previous *in vitro* studies, this may be the result of a lower quantity of isoflavone administration to the *in vitro* fermentation system. As previous studies utilized a minimum of 30 mg × L^-1^ of biochanin A to elucidate responses, the present study utilized 0.63 mg × L^-1^ of biochanin A, which may have not been enough to elicit a robust response. However, the current study was designed to examine effects of isoflavone administration on fermentation of ergot alkaloid-containing tall fescue, rather than effects on HAB and amino acid fermentation. Ergot alkaloid pressure on rumen fermentation was not clearly elucidated in the present study. VFA concentrations are indicative of rumen fermentation, which were not affected by each treatment. It is noteworthy that isoflavone administration did not alter VFA concentrations, and that seed type was the driving factor in any of the changes in VFA concentrations. Foote et al. [56] noted significant reductions in VFA flux and absorption in animals administered endophyte-infected tall fescue seed, lending support that nutrient utilization and energy absorption may be further delayed when cattle experience tall fescue toxicosis. As propionate supplies up to 65% of the glucose carbon in cattle [57], and livestock consuming endophyte-infected tall fescue produce reduced concentrations of VFAs [49], it is interesting that the acetate-to-propionate ratio tended to be reduced by endophyte-infected seed in the present study, as the combined effects of nutrient production, such as production of glucogenic precursors, and nutrient absorption will have significant impacts on animal production *in vivo*. The variability of this ratio may have been impacted by organisms identified in the current study that produce acetate, such as genera *Corynebacterium* and *Succinivibrio* [58, 59], as their respective abundances were similarly reduced with endophyte-infected seed. These microbiome-fermentation parameter associations hint at further evidence pertaining to reduced production and efficiency of cattle on endophyte-infected tall fescue. Further, Foote and colleagues reported similarly reduced VFA concentrations, when rumen buffers containing extracts of ergovaline were applied to heat stressed, ruminally cannulated steers [56]. The tendency for reduction of certain VFA concentrations observed in the present study, as well as reduced total VFAs described by Foote and colleagues [56], may provide further evidence that consumption of endophyte-infected tall fescue impacts nutrient production and absorption.

While rumen fermentation fluid bacterial populations varied among groups, the rumen microbiome is a dynamic ecosystem that is affected by various environmental and dietary factors, and thus changes in the bacterial taxa among groups may not be solely indicative of treatment with fescue seed or isoflavone treatment. As other studies have noted specific bacterial species capable of ergovaline degradation and benefits from isoflavones, further research should be conducted to validate consistent shifts in the rumen microbiome from ergot alkaloid pressure. It must also be noted that isoflavones are estrogenic. In finishing steers or cattle intended for harvest, this is of little issue and poses no risk to human health. However, the reproductive effects of isoflavone supplementation must be accounted for when administered in studies and systems where reproductive status is critical.

## Conclusions

In the current study, aNDF, ADF, and CP disappearance, as well as the abundances of 36 bacterial taxa, were impacted by isoflavone administration *in vitro*. Future research should be conducted to determine the impact of isoflavones on beef production *in vivo*, as the combined effects on ruminal feedstuff fermentation and HAB provide opportunities to naturally improve ruminal fermentation and enhance the efficiency of nutrient use in cattle.
